# Association between motor skills and executive function of children with autism spectrum disorder in Taiwan and the United States

**DOI:** 10.3389/fpubh.2023.1292695

**Published:** 2024-01-05

**Authors:** Ming-Chih Sung, Megan M. McClelland, William Massey, Samuel W. Logan, Megan MacDonald

**Affiliations:** ^1^Department of Human Performance and Health, University of South Carolina Upstate, Spartanburg, SC, United States; ^2^College of Health, Oregon State University, Corvallis, OR, United States

**Keywords:** motor abilities, cognitive function, preschool children, cross-cultural study, autism

## Abstract

**Objective:**

The purpose of this study was to examine the relationship between parent ratings of motor skills and executive function (EF) in children with autism spectrum disorder (ASD) in the United States and Taiwan.

**Materials and method:**

One hundred and seventy-two parents/legal guardians of children (4–6 years and 11 months old) with ASD were recruited from two countries, Taiwan (*n* = 100) and the United States (*n* = 72). The parents or guardians of the child with ASD completed a questionnaire including demographic information, child’s motor skills (using Children Activity Scale – Parents, ChAS-P), and child’s EF (using Childhood Executive Functioning Inventory, CHEXI). A series of hierarchical multiple regressions were conducted to determine whether ChAS-P (total motor score, fine motor skills, and gross motor skill) was associated with CHEXI (total EF score, working memory, and inhibition), after controlling for covariates (i.e., age, gender, race, body mass index, whether children received physical activity or cognitive training, parental education level).

**Results:**

Total motor skills, fine motor skills, and gross motor skills were significantly associated with EF in both working memory and inhibition as rated by parents in both countries (*β* = 0.21–0.57, *p* < 0.01), with the exception of a non-significant association between parent-rated total motor skills, fine motor skills, and gross motor skills, and inhibition among Taiwanese children with ASD. In addition, the associations between parent ratings of motor skills (i.e., fine motor and gross motor skills) and EF (i.e., working memory and inhibition) were similar between the two countries.

**Conclusion:**

Positive associations with specific aspects of parent ratings of fine motor and gross motor skills and working memory and inhibition were found in children with ASD from both countries. These findings have implications for future interventions and programs focused on improving early motor skills and EF development for young children with ASD from Taiwan and the United States.

## Introduction

Early childhood is a crucial period for the holistic development of a child’s social, emotional, cognitive, and physical needs to build a solid and broad foundation for lifelong learning and wellbeing (1). Young children above 5 years old experience considerable environmental changes as they move from preschool or home-based care into a more formal school setting like kindergarten. These changes include that young children interact with peers and teachers and are introduced to structured activities and curriculum, which will increase demands on their social, motor, and executive function (EF) skills (2). For many young children, this transition goes well, but it can be quite challenging and stressful for others, especially those children with autism spectrum disorder (3).

Autism spectrum disorder (ASD), a neurodevelopmental disorder, is defined by deficits in social communication and the presence of restricted or repetitive behaviors (4). Based on the recently revealed estimate from the Center for Disease Control and Prevention (CDC), the prevalence of ASD in 2020 increased from 1 in 68 to 1 in 36 children (5, 6). Similarly, individuals who identified with ASD in Taiwan increased from 10,160 to 15,750 between the years of 2010–2020 (7). Furthermore, the number of children aged 3 through 5 years served under the Individuals with Disabilities Education Improvement Act (IDEA) Part B services within the ASD category increased from 7.8 percent in 2012 to 10.8 percent in 2017 (8). The drastic increases in autism prevalence worldwide highlight the growing need for health, education, and social services for this population.

In addition to the core characteristics of ASD, deficits in motor skills have been consistently revealed in research on children with ASD (9, 10). “Motor skills” in the present study are defined using the term motor competence, which reflects various global terminologies (i.e., motor proficiency, motor performance, fundamental motor skill, and fine and gross motor skills) to describe goal-directed human movement (11). A myriad of studies have indicated that children with ASD demonstrate impaired or delayed motor skills, including postural control, motor coordination, and fine and gross motor skill (12–15). Evidence has suggested that 87% of children with ASD demonstrated significant motor impairment (16). Landa & Garrett-Mayer (17) indicated that children at higher risk for ASD at 14 months of age demonstrated evident motor skills deficits compared with peers without ASD. A recent meta-analysis further echoed Landa and Garrett-Mayer’s (17) hallmark research and indicated that infants with ASD exhibited motor behavior deficits early on, compared to infants without ASD, and this difference between the two groups amplified as age increased (18). Thus, it is essential to evaluate motor skills among young children with ASD and identify approaches to mitigate this developmental deficit.

Another commonly impaired developmental area in children with ASD is EF (19, 20). EF refers to a set of higher-order cognitive processes necessary for goal-directed behavior, including inhibitory control, cognitive flexibility, and working memory (21). EF deficits have consistently been reported in children with ASD (22–25). Research has shown that children with ASD demonstrated EF deficits in the performance of planning, inhibition of responses, and self-monitoring compared with their peers without ASD matched on IQ and language level (26). EF is critical to everyday functioning in life (27). If children with ASD experience deficits in EF, it might lead to difficulty in social interaction and quality of life. For example, inhibition, children may not be able to inhibit themselves and show aggressive behavior or distract easily in the class; cognitive flexibility, children may have problems shifting gears and thinking about things in different ways; working memory, children may not be able to hold on and visualize the numbers the teacher has called out. Further, EF deficits observed in individuals with ASD can also result in difficulties later in life, including independent behavior and work functioning (28, 29). Therefore, identifying and assessing EF impairments early in life to prevent long-term difficulties in children with ASD across a range of important functional domains is crucial.

In the past, motor skills and EF were regarded as two different constructs developing independently and discussed separately (30). Recent evidence, however, has indicated that these two constructs are interrelated (31, 32). For example, one study found that visual-motor integration skills of preschoolers aged 3 to 5 years significantly predicted their EF skills 7 months after (33). In addition, a systematic review of 21 studies suggested that complex motor skills, which were categorized to have a higher cognitive demand, demonstrated the strongest associations with higher-order cognitive skills (i.e., EF) among children without ASD (34). In the ASD population, evidence for the relationship between motor skills and EF has only been found in a couple of studies (35, 36). Schurink et al. (36) indicated that fine motor skills and balance were significantly correlated with cognitive flexibility among school-aged children with ASD. While the research mentioned above has indicated promising results, these studies have mainly focused on children in western countries such as Europe and the United States. Thus, how these relationships persist or differ in other countries and regions is important to understand.

Theoretical frameworks and neurobiological evidence provide the fundamental viewpoint of the co-occurrence and relationship between motor skills and cognitive development (37–39). The theoretical framework of learning to learn proposes that motor behaviors play an essential role in early learning (40). Within this framework, infants demonstrate their abilities to discover new solutions to solve novel problems through their motor flexibility when exploring and interacting with their surroundings. Thus, within this framework, early motor behaviors set the foundation for cognitive development and higher-order cognitive process (i.e., EF). In addition, research has shown that the pre-frontal cortex and cerebellum are co-activating while individuals are performing cognitive and motor tasks (41). Furthermore, the peak developmental age of both motor and cognitive skills in early childhood is around the same timeframe between the ages of 5 to 10 years (42). Therefore, examining the relationships between motor skills and EF early is critical given the evidence of theorized and the neurocognitive associations between the two domains.

Given that both motor and EF development are known to be influenced by the cultural context in which children grow up (43), a more comprehensive understanding of potential cross-cultural similarities or differences in motor skills and EF in children with ASD may significantly contribute to the global perspective on these critical aspects of development, particularly in the context of both Western and Eastern cultures. It is important to note that existing research has revealed noteworthy variations in motor skills and EF between children without ASD in Western and Eastern countries (44, 45). In motor skills comparisons, for instance, Chow et al. (44) utilized a performance-based motor skills assessment and found that Chinese children exhibited better fine motor skills performance, while American children demonstrated superior object control skills. In another study, the motor skills of 255 preschool children aged 4 to 6 from Hong Kong and 544 from Taiwan were assessed, and their performance on the Movement Assessment Battery (MABC) was compared to the standardized data from American children of the same age. The findings revealed statistically significant differences in MABC scores among typically developing children from Hong Kong, Taiwan, and the Unites States, indicating that Chinese children exhibited poorer performance (46).

Cross-cultural differences in EF also exist, Schmitt et al. (47) reported that Chinese children displayed higher EF performance at the outset of preschool compared to their American counterparts. Additionally, another study examined the EF abilities of 119 Chinese and 139 American typically developing children aged 4–5 years. The assessment included tasks such as Head-Toes-Knee-Shoulders (testing EF in a behavioral task), Sentence Completion task (evaluating working memory), and Woodcock-Johnson Pair Cancellation task (measuring attentional control). The results of this study indicated that young Chinese children demonstrated superior performance in behavioral regulation and attentional control tasks when compared to their American counterparts, while the performance in working memory tasks was similar for both groups (45). Similarly when the EF of preschoolers in China (*n* = 109) and in the United States (*n* = 107), were assessed, results indicated that the Chinese preschoolers exhibited better performance than their American counterparts across all the EF tasks (48). Despite these findings among children without ASD, it is unclear whether analogous patterns are observed among children with ASD, who are known to exhibit deficits in these domains, across different cultural backgrounds.

The delineation of a particular motor cognitive relation has the potential to inform earlier identification and inform key intervention initiatives, especially for young children with ASD. Although there has been a surge of research on the link between motor skills and EF in children without ASD (49–51), few researchers have examined the association between motor skills and executive functioning in young children with ASD (52, 53). Specifically, few if any studies have examined the cross-cultural similarities and differences in such relationships in young children with ASD. Therefore, the purpose of this study was to examine the relationship between parent ratings of motor skills and EF in children with ASD in the US and Taiwan to identify similarities and differences of associations between motor skills and EF among children with ASD across countries. The participants’ age range of 4–6 years and 11 months was chosen as it falls within the preschool period, which is a critical time for early intervention, particularly for children with ASD who are known to have deficits in both motor skills and EF. This age range provides a focused and homogeneous sample, minimizing the potential influence of additional factors that may come into play when children transition to elementary school settings. By concentrating on the preschool years, we aimed to capture the early developmental trajectory of motor skills and EF, as interventions during this period can significantly impact a child’s future outcomes. Using a cross-cultural sample of young children with ASD from Taiwan and the US, the present study provided important insights into cross-cultural universality and cultural variation in the links between motor skills and EF, especially in the autism community. Because there are few existing cross-cultural studies of the association between motor skills and EF, especially no studies in ASD children, the specific hypotheses regarding cross-cultural similarities and differences were developed based on the broader literature, including cross-cultural studies on children without ASD. It was hypothesized that (1) there would be significant associations between parent ratings of motor skills and EF in children with ASD from Taiwan and the US, respectively, and (2) the relationship between parent ratings of motor skills and EF in children with ASD from Taiwan would be stronger than the US.

## Materials and methods

### Sample

One hundred and seventy-two parents/legal guardians of children with ASD were recruited from two countries, Taiwan (*n* = 100) and the US (*n* = 72). Inclusion criteria of the present study included being a parent/guardian of a child with ASD and the child’s: (1) current aged between 4–6 years and 11 months, and (2) parental report of their child having a diagnosis of ASD, Pervasive Developmental Disorder-Not Otherwise Specified (PDD-NOS), or Asperger syndrome. Several strategies were employed to recruit parents/guardians of children with ASD. First, social media was used to reach potential participants, including personal social media websites (e.g., Facebook and Instagram), websites and social media pages of ASD-organization and associations in Taiwan and the US, and through Facebook advertisements (e.g., paid based on geographic region). These identified websites were asked to share a pre-established flyer/message to their social media page, which includes a link to the study survey. Second, flyers and bulletins with a QR code of the survey link were sent to targeted programs, ASD support groups, disability organizations, and pediatric services both in Taiwan and the US. Lastly, a research panel was purchased from “Centiment” due to the difficulty in recruiting enough US samples.^1^ Centiment was chosen because of their reputation and experience in surveying respondents that are difficult to reach, and Centiment has been used in previous studies (54, 55). Statistical analyses were conducted to compare the differences between the panel sample and the other US sample. Results showed no statistically significant differences in demographic information (e.g., child age, gender, race, body mass index (BMI), whether children received physical activity or cognitive training, parental education level) and outcome variables (e.g., total motor score, fine motor skills, gross motor skills, total EF score, working memory, and inhibition). Therefore, the two US samples were combined for further analyses.

### Measures

A comprehensive online survey distributed through the Qualtrics survey system (Provo, UT; https://www.qualtrics.com) was utilized in the present study and included three sections: (a) demographic information, (b) child motor skills, and (c) child EF. The English version of the survey was used by the American participants, and the Chinese version was used for participants in Taiwan.

### Demographic questionnaire

A demographic questionnaire was filled out by the parent/legal guardian of the child with ASD. The questions included participant and family background information, such as child age, gender, race/ethnicity, height, and weight, whether they received physical activity (e.g., after school physical activity program, soccer, or Taekwondo) and cognitive interventions or programs (e.g., physical therapy or occupational therapy), parent/guardian age, gender, race/ethnicity, living in urban/rural area, educational level, and annual income.

### Motor skill questionnaire

The motor skills were measured by the subitems of the Children Activity Scale – Parents (ChAS-P) in this study. ChAS-P is an efficient and appropriate parent-proxy questionnaire measuring the gross and fine motor skills and activities of daily living of children aged 4–8 years during everyday functional/play skills in a natural environment (56). The time for parents/guardians to complete ChAS-P is about 5 min. The questionnaire asks parents to evaluate their child’s motor skills or activity of daily living by comparing their child’s performance to another child. ChAS-P consists of 27 questions with a 5-point Likert scale ranging from 5 = “less adequately,” 4 = “adequately,” 3 = “almost well,” 2 = “well,” and 1 = “very well.” These 27 questions are grouped into four factors: gross motor skills (e.g., maintaining balance, playing in the playground), fine motor skills (e.g., writing/copying shapes, drawing), organization in space and time (representing the ability to organize movement in time and space, e.g., organizing self in preparation for going out), and activities of daily living (e.g., eating without getting dirty, self-dressing). Scores are summed for a total score ranging from 27 (lowest) to 135 (highest), with lower scores rated by parents reflecting better motor skills among children. Given the aims of the current study, fine motor skills (6 items) and gross motor skills (6 items) were used for the analyses of this study. The summary scores from motor skill subitems range from 12 (lowest) to 60 (highest).

The ChAS-P was selected for use because it has demonstrated good internal consistency, construct validity, and concurrent validity, with a significant moderate correlation between the Movement Assessment Battery (MABC) and ChAS-P (*r* = 0.51, *p* < 0.001) (56); in addition this assessment was free for use. Further, the ChAS-P has been used for measuring the motor performance of children with other developmental disorders, such as attention-deficit/hyperactivity disorder (ADHD) (57) and developmental coordination disorder (DCD) (58), and was recommended by Bardid et al. (59) as an appropriate parent proxy for measurement of motor skills when direct individually administered measures are not feasible. The author translated English version of ChAS-P to Chinese version based on the cross-cultural adaptation of instruments.

### Executive function questionnaire

Childhood Executive Functioning Inventory (CHEXI) was employed to measure problems with EF. The CHEXI (60) is a 24-item parent-report inventory that assesses the behavioral manifestations of EF abilities in children aged 4 to 12 years. The CHEXI is an open-access tool with multiple language versions, including Chinese.^2^ The administration time of CHEXI is about 5 min for parents/ guardians to complete the form. CHEXI capitalizes on observations of children in their naturalistic settings to quantify their EF impairments during participation in regular life activities. The CHEXI is comprised of 24 questions with a 5-point Likert scale ranging from 1 being “Definitely not true” to 5 being “Definitely true.” The example items for the working memory subscale include “Has difficulty with tasks or activities that involve several steps” and “Has difficulty remembering lengthy instructions.” An example item for the inhibition subscale includes “Has difficulty holding back his/her activity despite being told to do so.” Parents/ guardians will read such a statement and indicate how well that statement is true for the child. Based on the original study conducted by the creator of the CHEXI, the questionnaire items naturally clustered into two factors when administered to young children in kindergarten (60). These factors were identified as ‘working memory,’ which encompassed the working memory (11 items) and planning (4 items) subscales, and ‘inhibition,’ which included the regulation (5 items) and inhibition (6 items) subscales, according to the guidelines provided in the questionnaire’s instructions. Each question’s scores are summed for a total score ranging from 24 being the lowest to 120 being the highest, with higher scores indicating greater EF problems.

The reason for using the CHEXI to measure EF among children with ASD was because it provides the measurement of a child’s EF deficits in the context of everyday demands as rated by parents. Research has indicated that neuropsychological tests administered in a lab may not be representative of the more complex daily lives of children (61). In addition, many lab-based EF tests have limits in their ecological validity and generalizability (62). Research also revealed that CHEXI has higher discriminant validity than the one found in neuropsychological tests (63). A recent meta-analysis study indicated that parent-reported ratings of EF had larger effect sizes compared to psychometric tests or experimental tasks (23). Thorell & Nyberg (60) suggested that questionnaires reported by raters capture the child’s behavior in the real world based on observations during an extended period. Also, evidence has shown that both English and Chinese version of CHEXI demonstrated good validity and reliability (64, 65). Further, CHEXI has been used in children with developmental disorders such as ADHD (63), and in typically developing young Taiwanese children (65).

### Procedure

Ethical approval for the study was received from the Institutional Review Board at Oregon State University. In both the US and Taiwan, participants were recruited from various ASD organizations, pediatric services and schools, social media websites, and advertisements (e.g., Facebook). These identified websites were asked to post to their social media page with a link to the survey. The messages were preconstructed, minimizing the work for the organizations and maximizing consistency. The time for completing the demographic questionnaire, ChAS-P, and CHEXI is usually 5 min for each (total about 15 min). As much as possible, parents/guardians were encouraged to complete the surveys in a non-distracting environment.

### Statistical analysis

Descriptive statistics, including means and standard deviations, were computed for demographic information (e.g., age, gender, race, BMI, IEP, whether children received physical activity or cognitive training, living area, parents/guardians age, parental education level, and annual household income), ChAS-P, and CHEXI scores. In this study, we collected a comprehensive set of demographic information from participants. These variables were chosen based on prior research indicating their potential influence on motor skills and EF, which are the primary outcome variables of interest.

We also conducted several preliminary tests to determine the most relevant covariates prior to our main analyses. Chi-square tests of independence were conducted to assess associations between categorical demographic variables, including gender, IEP, whether children received physical activity or cognitive training, living area, parental educational level, and annual household income. Independent t-tests were performed to examine potential differences in continuous outcome variables, including age, BMI, and parents/guardians age, between participants from the two countries. In cases where statistical significance was observed, the variables were included as covariates in subsequent regression analyses. It is important to note that, even when certain variables such as age and gender did not exhibit statistical significance in the initial chi-square or t-tests, they were retained as covariates in the regression model. This decision was made based on theoretical considerations, acknowledging the possibility of their latent impact on the outcomes of interest. We recognize the importance of thorough covariate selection and have taken this into account in our analytical approach.

The following outcomes of ChAS-P were used for analysis: (1) fine motor skills, (2) gross motor skills, and (3) total motor score. For the variables in EF, (1) working memory, (2) inhibition, and (3) total EF score in CHEXI were used for analysis. Previous research examining the associations between motor skills and EF in children with and without disabilities had mixed findings. While the majority of studies indicated that fine motor skills were associated with EF (31, 34, 66), some studies found associations between gross motor skills and EF (67–69). Due to the inconsistent findings in the previous research, our goal was to investigate the specific relationships between parent ratings of motor skills and EF in children with ASD in the United States.

Thus, to investigate these associations in children with ASD from Taiwan and the United States, separate hierarchical linear regressions were conducted.

In our hierarchical regression strategy, we systematically evaluated the relationship between parent ratings of motor skills and EF in children with ASD in both the United States and Taiwan. To ensure a comprehensive assessment while addressing potential multicollinearity issues, a two-block hierarchical linear regression approach was employed. In Block 1 of the regression models, we included all covariates that were deemed relevant to the analysis, including age, gender, BMI, whether children received physical activity or cognitive training, and parental education level. The criteria for variables assessed in Block 1 to be carried forward into Block 2 were based on their theoretical relevance to the analysis and their potential influence on the relationships under investigation. In Block 2 of the hierarchical regressions, we introduced one of the motor skills variables as the independent variable, and calculated separate regression analyses for: total motor skills, fine motor skills, and gross motor skills. This step was taken separately for participants from Taiwan and the United States, resulting in a total of 12 hierarchical linear regressions (six for participants from Taiwan and six for participants from the United States). Hierarchical regressions were employed to determine whether the independent variables (i.e., motor skills) accounted for a statistically significant portion of the variance in the dependent variable (i.e., EF) after accounting for all other covariates.

To examine whether children with ASD from Taiwan and the US showed different relationships between motor skills and EF, another hierarchical regression analysis was employed, with EF as the dependent variable. All covariates were entered in Block 1, and the total motor skills, country, and the interaction term of total motor skills x country were entered in Block 2. The purpose of this interaction term was to assess whether there were differences in the relationship between parent ratings of motor skills and EF in children with ASD from Taiwan and the United States. The ‘country’ was treated as a categorical variable, with two levels representing the two countries in our study, Taiwan and the United States. All statistical analyses were conducted using RStudio (version 3.6.1). An alpha level of.01 was used for all statistical tests. In this study, a more stringent alpha level for determining statistical significance was adapted, setting it at.01 instead of the conventional.05. This modification was to minimize the risk of Type I errors, especially in light of the 13 hierarchical linear regressions conducted as part of our analysis. By employing this adjusted alpha level, we aimed to control the familywise error rate across multiple tests.

## Results

### Descriptive analysis

Descriptive statistics for participants from Taiwan and the US are presented in Table 1, including means, standard deviations for continuous variables, and proportions for categorical variables. Statistical differences at the 5% significance level were observed in several demographic variables: (1) whether children with ASD received cognitive training. Children with ASD from Taiwan had a higher percentage compared to their peers from the US; (2) parental education level. Parents from Taiwan generally had higher degrees compared to parents from the United States; (3) BMI. Children with ASD from Taiwan had lower BMI compared to their peers from the US; (4) parents’ age. Parents from Taiwan were older than parents from the United States (see Table 1).

**Table 1:** Characteristics of participants.

	Taiwan (*n* = 100)	USA (*n* = 72)	
	Mean (SD)/ Proportion	Mean (SD)/ Proportion	*p*
Age	4.99 (0.80)	5.00 (0.73)	0.41
4 years old	*n* = 32 (32.0%)	*n* = 19 (26.4%)	
5 years old	*n* = 37 (37.0%)	*n* = 34 (47.2%)	
6 years old	*n* = 31 (31.0%)	*n* = 19 (26.4%)	
Gender			0.25
Boys	*n* = 81 (81.0%)	*n* = 53 (73.6.%)	
Girls	*n* = 19 (19.0%)	*n* = 19 (26.4%)	
Race			
Taiwanese	*n* = 96 (96%)		
Taiwanese aborigines	*n* = 4		
White/Caucasian		*n* = 39 (52.8%)	
Black/African American		*n* = 9 (12.5%)	
Hispanic/Latino		*n* = 12 (16.7%)	
Asian		*n* = 8 (11.1%)	
Native American		*n* = 2	
Other		*n* = 2	
Prefer not to say		*n* = 1	
BMI	15.89 (2.25)	19.72 (6.61)	< 0.001
IEP			0.92
Yes	*n* = 71 (71.0%)	*n* = 50 (69.4%)	
No	*n* = 27 (27.0%)	*n* = 21 (29.2%)	
Prefer not to say	*n* = 2	*n* = 1	
(Adapted) Physical Activity			0.06
Yes	*n* = 51 (51.0%)	*n* = 28 (38.9%)	
No	*n* = 49 (49.0%)	*n* = 42 (58.3%)	
Prefer not to say	*n* = 0	*n* = 2	
Cognitive Training			< 0.001
Yes	*n* = 76 (76.0%)	*n* = 31 (43.1%)	
No	*n* = 24 (24.0%)	*n* = 38 (52.8%)	
Prefer not to say	*n* = 0	*n* = 3	
Living Area			0.15
Rural	*n* = 18 (18.0%)	*n* = 20 (27.8%)	
City	*n* = 77 (77.0%)	*n* = 51 (70.8%)	
Prefer not to say	*n* = 5	*n* = 1	
Parents/guardians Age	38.79 (4.84)	35.36 (6.14)	< 0.001
Parental Education Level			< 0.001
Elementary school	*n* = 1	*n* = 1	
High school	*n* = 8	*n* = 23 (31.9%)	
College (2 years)	*n* = 6	*n* = 15 (20.8%)	
College (4 years)	*n* = 53 (53.0%)	*n* = 22 (30.6%)	
Master degree	*n* = 31 (31.0%)	*n* = 10 (13.9%)	
Ph.D. degree	*n* = 1	*n* = 1	
Annual household income			0.20
Less than $20,000	*n* = 7	*n* = 7	
$20,000 to $34,999	*n* = 20 (20%)	*n* = 8	
$35,000 to $49,999	*n* = 13 (13%)	*n* = 18 (25.0%)	
$50,000 to $74,999	*n* = 21 (21%)	*n* = 12 (16.7%)	
$75,000 to $99,999	*n* = 12 (12%)	*n* = 13 (18.1%)	
$100,000 or more	*n* = 19 (19%)	*n* = 11 (15.3%)	
Prefer not to say	*n* = 8	*n* = 3	

BMI, body mass index; IEP, individualized education program.

The descriptive statistics for motor skills (total motor skills scores, fine motor skills, and gross motor skills) and EF (total EF scores, working memory, and inhibition) were presented in Table 2. The focus of the analyses was not to make comparisons between countries in motor skills and EF. Instead, our aim was to identify similarities and differences across countries in terms of associations between motor skills and EF.

**Table 2:** Mean and standard deviation of outcome variables.

	Taiwan (*n* = 100)	USA (*n* = 72)
	Mean (SD)	Mean (SD)
Motor skills		
Total motor skills	40.12 (11.12)	33.53 (12.54)
Fine motor skills	21.78 (6.35)	18.03 (6.96)
Gross motor skills	18.34 (6.58)	15.50 (6.60)
Executive function		
Total executive function	86.31 (15.51)	84.51 (16.93)
Working memory	45.42 (9.92)	44.67 (10.16)
Inhibition	40.89 (6.66)	39.85 (8.12)

Higher the scores in motor skills and executive function mean more deficits.

### Analytic strategy

It is important to note that the results from the hierarchical regression analyses presented below were conducted separately for children with ASD from Taiwan and the United States to explore the relationships within each cultural context. This approach allowed us to gain insights into the associations between motor skills and EF while considering the unique characteristics within each group.

### Working memory

The hierarchical regression analysis indicated that Taiwanese children with ASD had parent ratings of total motor skills that were significantly related to ratings of working memory after controlling for covariates (*β* = 0.39, *p* < 0.001). Overall, the model including Taiwanese children with ASD explained nearly 28% of the variance in working memory (i.e., adjusted *R*^2^ = 0.28). American children with ASD had total motor skill ratings that were also significantly associated with ratings of working memory (*β* = 0.57, *p* < 0.001) after controlling for covariates. The model including American children with ASD accounted for nearly 31% of the variance in working memory (i.e., adjusted *R*^2^ = 0.31) (see Table 3).

**Table 3:** Hierarchical multiple regression analyses for total motor skills predicting working memory.

	Taiwan (*n* = 100)			US (*n* = 72)		
	Δ*R*^2^	*β*	*t*	Δ*R*^2^	*β*	*t*
Block 1	0.14^*^			−0.02		
Age		0.27^*^	2.83		−0.06	−0.52
Gender		−0.07	−0.69		−0.05	−0.43
BMI		−0.01	−0.14		−0.03	−0.24
PA		0.09	0.84		0.23	1.67
Cognitive		−0.25^*^	−2.63		−0.02	−0.16
Parental Edu		−0.20	−2.01		0.13	0.99
Block 2	0.28^**^			0.31^**^		
Age		0.22^*^	2.48		−0.05	−0.50
Gender		−0.03	−0.31		0.03	0.28
BMI		−0.06	−0.66		−0.02	−0.19
PA		0.10	1.072		0.12	0.99
Cognitive		−0.16	−1.83		0.07	0.57
Parental Edu		−0.17	−1.80		0.11	1.01
Total motor skills		0.39^**^	4.28		0.57^**^	5.61

BMI, body mass index; PA, whether a child received physical activity program/intervention (levels: ‘Yes,’ ‘No, “Prefer not to say’); Cognitive, whether a child received a cognitive training (levels: ‘Yes, “No, “Prefer not to say’); Parental Edu, parental education level (levels: ‘elementary school,’ ‘high school,’ ‘college (2 years),’ ‘college (4 years),’ ‘master’s degree,’ ‘PhD degree’). The negative Δ*R*^2^ for Block 1 indicates that the inclusion of covariates did not substantially improve the model’s ability to explain variance in the dependent variable.

^**^p < 0.01.

^*^p < 0.05.

The association between parent ratings of fine motor skills and working memory of Taiwanese children with ASD was significant in Block 2 of hierarchical regression analysis after controlling for covariates (*β* = 0.41, *p* < 0.001). Overall, the model including Taiwanese children with ASD explained nearly 29% of the variance in working memory (i.e., adjusted *R*^2^ = 0.29). In addition, the association between fine motor skills and working memory ratings of American children with ASD was also significant after accounting for covariates (*β* = 0.53, *p* < 0.001). The model including American children with ASD accounted for 24% of the variance in working memory (i.e., adjusted *R*^2^ = 0.24) as shown in Table 4.

**Table 4:** Hierarchical multiple regression analyses for fine motor skills predicting working memory.

	Taiwan (*n* = 100)			US (*n* = 72)		
	Δ*R*^2^	*β*	*t*	Δ*R*^2^	*β*	*t*
Block 1	0.14^*^			−0.02		
Age		0.27^*^	2.83		−0.06	−0.52
Gender		−0.07	−0.69		−0.05	−0.43
BMI		−0.01	−0.14		−0.03	−0.24
PA		0.09	0.84		0.23	1.67
Cognitive		−0.25^*^	−2.63		−0.02	−0.16
Parental Edu		−0.20	−2.01		0.13	0.99
Block 2	0.29^**^			0.24^**^		
Age		0.25^**^	2.84		−0.01	−0.06
Gender		−0.03	−0.39		0.06	0.57
BMI		−0.06	−0.73		−0.01	−0.06
PA		0.12	1.31		0.13	1.05
Cognitive		−0.13	−1.44		0.01	0.11
Parental Edu		−0.15	−1.59		0.10	0.90
Fine motor skills		0.41^**^	4.48		0.53^**^	4.85

BMI, body mass index; PA, whether a child received physical activity program/intervention (levels: ‘yes,’ ‘no,’ ‘prefer not to say’); Cognitive, whether a child received a cognitive training (levels: ‘yes,’ ‘no,’ ‘prefer not to say’); Parental Edu, parental education level (levels: ‘elementary school,’ ‘high school,’ ‘college (2 years),’ ‘college (4 years),’ ‘master’s degree,’ ‘PhD degree’). The negative Δ*R*^2^ for Block 1 indicates that the inclusion of covariates did not substantially improve the model’s ability to explain variance in the dependent variable.

^**^p < 0.01.

^*^p < 0.05.

After controlling for covariates, parent ratings of gross motor skills were significantly associated with working memory ratings among children with ASD from Taiwan (*β* = 0.26, *p* = 0.006). Overall, the model including Taiwanese children with ASD explained 20% of the variance in working memory (i.e., adjusted *R*^2^ = 0.20). Further, children with ASD from the US had gross motor skill ratings that were significantly related to ratings of working memory after controlling for covariates (*β* = 0.55, *p* < 0.001). The model including American children with ASD accounted for nearly 28% of the variance in working memory (i.e., adjusted *R*^2^ = 0.28) (see Table 5).

**Table 5:** Hierarchical multiple regression analyses for gross motor skills predicting working memory.

	Taiwan (*n* = 100)			US (*n* = 72)		
	Δ*R*^2^	*β*	*t*	Δ*R*^2^	*β*	*t*
Block 1	0.14^*^			−0.02		
Age		0.27^*^	2.83		−0.06	−0.52
Gender		−0.07	−0.69		−0.05	−0.43
BMI		−0.01	−0.14		−0.03	−0.24
PA		0.09	0.84		0.23	1.67
Cognitive		−0.25^*^	−2.63		−0.02	−0.16
Parental Edu		−0.20	−2.01		0.13	0.99
Block 2	0.20^*^			0.28^**^		
Age		0.23^*^	2.43		−0.10	−0.99
Gender		−0.04	−0.45		−0.03	−0.29
BMI		−0.03	−0.36		−0.04	−0.36
PA		0.08	0.82		0.14	1.13
Cognitive		−0.23^*^	−2.45		0.10	0.84
Parental Edu		−0.20	−2.02		0.12	1.13
Gross motor skills		0.26^**^	2.81		0.55^**^	5.29

BMI, body mass index; PA, whether a child received physical activity program/intervention (levels: ‘yes,’ ‘no,’ ‘prefer not to say’); Cognitive, whether a child received a cognitive training (levels: ‘yes,’ ‘no,’ ‘prefer not to say’); Parental Edu, parental education level (levels: ‘elementary school,’ ‘high school,’ ‘college (2 years),’ ‘college (4 years),’ ‘master’s degree,’ ‘PhD degree’). The negative Δ*R*^2^ for Block 1 indicates that the inclusion of covariates did not substantially improve the model’s ability to explain variance in the dependent variable.

^**^p < 0.01.

^*^p < 0.05.

### Inhibition

The hierarchical regression analysis showed that, under the more stringent alpha level of 0.01, the association between parent ratings of total motor skills and inhibition among Taiwanese children with ASD did not reach statistical significance after controlling for covariates (*β* = 0.26, *p* = 0.01). Overall, the model including children with ASD from Taiwan explained nearly 12% of the variance in inhibition (i.e., adjusted *R*^2^ = 0.12). American children with ASD had total motor skill ratings that were significantly associated with ratings of inhibition after accounting for covariates (*β* = 0.40, *p* < 0.001). The model including children with ASD from the US accounted for 14% of the variance in inhibition (i.e., adjusted *R*^2^ = 0.14), as shown in Table 6.

**Table 6:** Hierarchical multiple regression analyses for total motor skills predicting inhibition.

	Taiwan (*n* = 100)			US (*n* = 72)		
	Δ*R*^2^	*β*	*t*	Δ*R*^2^	*β*	*t*
Block 1	0.07^*^			−0.01		
Age		0.23^*^	2.31		−0.09	−0.71
Gender		−0.10	−1.03		−0.19	−1.53
BMI		−0.01	−0.10		0.05	0.43
PA		−0.03	−0.24		0.12	0.89
Cognitive		−0.20^*^	−2.03		0.13	0.87
Parental Edu		−0.14	−1.36		0.11	0.87
Block 2	0.12^*^			0.14^**^		
Age		0.20^*^	2.02		−0.08	−0.68
Gender		−0.08	−0.79		−0.13	−1.14
BMI		−0.04	−0.40		0.06	0.53
PA		−0.02	−0.15		0.04	0.32
Cognitive		−0.14	−1.46		0.19	1.41
Parental Edu		−0.12	−1.17		0.10	0.83
Total motor skills		0.26^*^	2.56		0.40^**^	3.51

BMI, body mass index; PA, whether a child received physical activity program/intervention (levels: ‘yes,’ ‘no,’ ‘prefer not to say’); Cognitive, whether a child received a cognitive training (levels: ‘yes,’ ‘no,’ ‘prefer not to say’); Parental Edu, parental education level (levels: ‘elementary school,’ ‘high school,’ ‘college (2 years),’ ‘college (4 years),’ ‘master’s degree,’ ‘PhD degree’). The negative Δ*R*^2^ for Block 1 indicates that the inclusion of covariates did not substantially improve the model’s ability to explain variance in the dependent variable.

^**^p < 0.01.

^*^p < 0.05.

When considering the fine motor skills of Taiwanese children with ASD, the association with inhibition remained non-significant in Block 2 of hierarchical regression analysis after controlling for covariates (*β* = 0.21, *p* = 0.04) under the more stringent alpha level, accounting for approximately 10% of the variance in inhibition (i.e., adjusted *R*^2^ = 0.10). However, the association between fine motor skills and inhibition ratings of American children with ASD remained significant after accounting for covariates (*β* = 0.39, *p* = 0.001). The model including children with ASD from the US accounted for nearly 13% of the variance in working memory (i.e., adjusted *R*^2^ = 0.13) (see Table 7).

**Table 7:** Hierarchical multiple regression analyses for fine motor skills predicting inhibition.

	Taiwan (*n* = 100)			US (*n* = 72)		
	Δ*R*^2^	*β*	*t*	Δ*R*^2^	*β*	*t*
Block 1	0.07^*^			−0.01		
Age		0.23^*^	2.31		−0.09	−0.71
Gender		−0.10	−1.03		−0.19	−1.53
BMI		−0.01	−0.10		0.05	0.43
PA		−0.03	−0.24		0.12	0.89
Cognitive		−0.20^*^	−2.03		0.13	0.87
Parental Edu		−0.14	−1.36		0.11	0.87
Block 2	0.10^*^			0.134^*^		
Age		0.22^*^	2.23		−0.04	−0.38
Gender		−0.09	−0.87		−0.10	−0.87
BMI		−0.04	−0.36		0.07	0.62
PA		−0.01	−0.07		0.04	0.34
Cognitive		−0.14	−1.36		0.15	1.14
Parental Edu		−0.11	−1.10		0.09	0.76
Fine motor skills		0.21^*^	2.07		0.39^**^	3.40

BMI, body mass index; PA, whether a child received physical activity program/intervention (levels: ‘yes,’ ‘no,’ ‘prefer not to say’); Cognitive, whether a child received a cognitive training (levels: ‘yes,’ ‘no,’ ‘prefer not to say’); Parental Edu, parental education level (levels: ‘elementary school,’ ‘high school,’ ‘college (2 years),’ ‘college (4 years),’ ‘master’s degree,’ ‘PhD degree’). The negative Δ*R*^2^ for Block 1 indicates that the inclusion of covariates did not substantially improve the model’s ability to explain variance in the dependent variable.

^**^p < 0.01.

^*^p < 0.05.

Similarly, after applying the stricter alpha level of 0.01, the relationship between parent ratings of gross motor skills and inhibition remained non-significant for Taiwanese children with ASD after controlling for covariates (*β* = 0.22, *p* = 0.03), explaining approximately 11% of the variance in inhibition (i.e., adjusted *R*^2^ = 0.11). Nonetheless, American children with ASD had gross motor skills ratings that were significantly associated with ratings of inhibition after accounting for covariates (*β* = 0.35, *p* = 0.003). The model including children with ASD from the US accounted for nearly 11% of the variance in inhibition (i.e., adjusted *R*^2^ = 0.11) (see Table 8).

**Table 8:** Hierarchical multiple regression analyses for gross motor skills predicting inhibition.

	Taiwan (*n* = 100)			US (*n* = 72)		
	Δ*R*^2^	*β*	*t*	Δ*R*^2^	*β*	*t*
Block 1	0.07^*^			−0.01		
Age		0.23^*^	2.31		−0.09	−0.71
Gender		−0.10	−1.03		−0.19	−1.53
BMI		−0.01	−0.10		0.05	0.43
PA		−0.03	−0.24		0.12	0.89
Cognitive		−0.20^*^	−2.03		0.13	0.87
Parental Edu		−0.14	−1.36		0.11	0.87
Block 2	0.11^*^			0.11^*^		
Age		0.19	1.96		−0.11	−0.96
Gender		−0.08	−0.84		−0.17	−1.5
BMI		−0.03	−0.27		0.05	0.41
PA		−0.03	−0.29		0.06	0.45
Cognitive		−0.18	−1.85		0.21	1.50
Parental Edu		−0.14	−1.34		0.11	0.90
Gross motor skills		0.22^*^	2.27		0.35^**^	3.06

BMI, body mass index; PA, whether a child received physical activity program/intervention (levels: ‘yes,’ ‘no,’ ‘prefer not to say’); Cognitive, whether a child received a cognitive training (levels: ‘yes,’ ‘no,’ ‘prefer not to say’); Parental Edu, parental education level (levels: ‘elementary school,’ ‘high school,’ ‘college (2 years),’ ‘college (4 years),’ ‘master’s degree,’ ‘PhD degree’). The negative Δ*R*^2^ for Block 1 indicates that the inclusion of covariates did not substantially improve the model’s ability to explain variance in the dependent variable.

^**^p < 0.01.

^*^p < 0.05.

The last regression model showed that the interaction term between motor skills and the country was not significant, indicating that the differences in associations between parent ratings of motor skills and EF did not vary as a function of the country (*β* = 0.06–0.09, *p* = 0.64–0.83) (see Table 9). In other words, the relation between motor skills and EF ratings was not significantly different in children with ASD from Taiwan compared to the children with ASD from the United States.

**Table 9:** Hierarchical multiple regression analyses for motor skills predicting EF (Combined data from Taiwan and the United States, *n* = 172).

	Total EF			Working memory			Inhibition		
	Δ*R*^2^	*β*	*t*	Δ*R*^2^	*β*	*t*	ΔR^2^	*β*	*t*
Block 1	0.03			0.04^*^			0.01		
Age		0.14	1.89		0.16^*^	2.04		0.10	1.34
Gender		−0.11	−1.48		−0.07	−0.92		−0.15^*^	−1.99
BMI		−0.02	−0.24		−0.04	−0.47		0.01	0.11
PA		0.16	1.94		0.19^*^	2.40		0.08	1.00
Cognitive		−0.14	−1.69		−0.18^*^	−2.18		−0.06	−0.76
Parental Edu		−0.05	−0.64		−0.07	−0.86		−0.02	−0.24
Block 2	0.23^**^			0.27^**^			0.17^**^		
Age		0.11	1.61		0.12	1.80		0.08	1.07
Gender		−0.05	−0.78		−0.01	−0.12		−0.11	−1.45
BMI		−0.01	−0.18		−0.04	−0.58		0.03	0.34
PA		0.12	1.59		0.15^*^	2.12		0.05	0.63
Cognitive		−0.05	−0.60		−0.09	−1.16		0.02	0.21
Parental Edu		−0.06	−0.75		−0.07	−0.89		−0.04	−0.45
Country		0.07	0.82		0.10	1.33		0.00	0.04
Total motor skills		0.43^**^	4.32		0.47^**^	4.85		0.30^*^	2.86
Total motor skills*Country		0.08	0.79		0.06	0.65		0.09	0.83

BMI, body mass index; PA, whether a child received physical activity program/intervention (levels: ‘yes,’ ‘no,’ ‘prefer not to say’); Cognitive, whether a child received a cognitive training (levels: ‘yes,’ ‘no,’ ‘prefer not to say’); Parental Edu, parental education level (levels: ‘elementary school,’ ‘high school,’ ‘college (2 years),’ ‘college (4 years),’ ‘master’s degree,’ ‘PhD degree’). The negative Δ*R*^2^ for Block 1 indicates that the inclusion of covariates did not substantially improve the model’s ability to explain variance in the dependent variable.

^**^p < 0.01.

^*^p < 0.05.

## Discussion

The purpose of this study was to examine the relationships between parent ratings of motor skills and EF in children with ASD from the United States and Taiwan. Specifically, this study aimed to answer (1) what is the relationship between motor skills and EF ratings in young children with ASD from Taiwan and the US and (2) how do such relationships in children with ASD in Taiwan differ from the children with ASD in the US? Results indicated that parent ratings of total motor skills, fine motor skills, and gross motor skills were significantly associated with EF in both working memory and inhibition in both countries. However, non-significant associations between parent-rated total motor skills, fine motor skills, and gross motor skills, and inhibition among Taiwanese children with ASD were observed under a more stringent alpha level. Another important finding was that considerable similarities were revealed between Taiwan and the US children with ASD in the relationships between ratings of motor skills and EF. This is one of the first studies, to the authors’ knowledge, investigating the associations between motor skills, including both fine and gross motor skills, and EF, including working memory and inhibition, in young children with ASD across two countries.

Findings indicated that the significant associations between ratings of motor skills and EF in children with ASD did not depend on country, suggesting that these relationships are culturally comparable, with significant and positive correlations of magnitude in both countries. No research, to date, has explored the link between motor skills and EF in young children with ASD cross-culturally. It might be possible that the relation between motor skills and EF follow the same developmental timeframe and trajectory, regardless of the different contextual influences, such as geographical, cultural, and educational factors. While the exploratory nature of this study warrants future cross-cultural research, the current findings partially corroborate evidence from previous research on children with ASD in western countries (36, 52, 70). Schurink et al. (36) found significant relationships between manual dexterity, balance, and planning ability measured by objective assessments among children with PDD-NOS, a type of ASD, indicating that inferior motor skills performance is associated with poorer EF. Such a relationship may be explained by considering that substantial comorbidity between deficits in motor skills and cognitive functioning was observed in children with neurodevelopmental disorders. Indeed, several studies have suggested that the relations between motor skills and cognitive development were manifested in children with intellectual disabilities (71–73), Down syndrome (74), developmental coordination disorder (75) and attention-deficit hyperactivity disorder (76). Recently, one study utilized objective assessments to examine the relationship between EF, particularly attention and impulse control, and motor function in 15 school-aged children with ASD aged 8–14 years in the US. The findings revealed significant associations between EF and motor functions in children with ASD (77). In addition, Kim and colleagues (52) identified that fine motor skills, as opposed to gross motor skills, were predictive of cognitive skill enhancements after adjusting for demographic variables and initial skill levels in a cohort of pre-kindergarten American children with developmental disabilities, including those with ASD. It’s crucial to note the methodological differences between these studies and the present research. While the aforementioned studies relied on objective assessments, our study utilized subjective parent reports. Additionally, the age range of participants in our study is different from the studies above. As a result, when interpreting the findings and considering their implications, it is important to exercise caution and take into account these variations. Although the adoption of a stricter alpha level (i.e., 0.001) led to the non-significance of certain correlations between motor skills and EF in our study, it is noteworthy that the effect sizes of our findings remain consistent with those observed in the studies mentioned earlier (52, 77), demonstrating moderate relationships between motor skills and EF among children with ASD. Thus, the present study contributes to a greater magnitude of existing literature in the field of disability, indicating the relationship between motor skills and two domains of EF (i.e., working memory and inhibition) among young children with ASD from different countries.

The current findings are in accordance with the theoretical framework of learning to learn (40) and embodied cognition theory (39), which suggests that cognition develops as a result of an agent’s bodily interactions with their surroundings. For instance, children develop the capacity for problem-solving through the interaction of their motor behavior and exploring and interacting with the environment. With this, early motor skills seem to lay the foundation for later cognitive development among children. The theoretical perspective was further supported by neuroimaging research (41). Empirical evidence has revealed that the rostral premotor cortex connects between motor and cognitive networks and that brain regions previously thought to be involved only in motor activities (i.e., cerebellum and basal ganglia) or cognitive activities (i.e., the prefrontal cortex) are co-activating while people engage in certain cognitive and motor tasks. (41, 78). Moreover, previous evidence has revealed that motor and cognitive development are highly associated and further suggested that motor behaviors that facilitate interaction with the environment during early childhood are critical for cognitive growth (79). Our findings further reinforce the theorized and neuroimaging evidence on the associations between motor skills and EF in the ASD population.

### Motor skills and working memory

Consistent with our hypothesis, the findings of the present study showed that both parent ratings of fine and gross motor skills were significantly related to working memory ratings in children with ASD from Taiwan and the US. Our results corroborate previous studies revealing that fine motor skills are associated with working memory in children at-risk/with ASD (80, 81) and preschool-aged children without ASD (72). Rosenblum et al. (80) suggested a significant relationship between handwriting and working memory among school-aged children with ASD. Authors assumed that handwriting, especially in the context of story-writing, might be difficult and particularly affected by even minor distractions for children with ASD, who are known to have deficits in working memory. Another plausible explanation might be the fact that working memory is needed for various activities involving fine motor skills, especially visual-motor integration (33). The items evaluating fine motor skills in the current study consisted of how well a child does in using scissors for cutting, in the constructive play and creative activities (e.g., Lego). These complex motor tasks, such as building blocks or manipulating scissors to cut along a line, likely involve the processes of working memory to control the coordination necessary to complete the activity/task successfully (34). Indeed, children spend a significant amount of time engaged in fine motor skills such as drawing, cutting, folding, and manipulating objects in preschool settings (82, 83). These activities have certain demand on fine motor skills and visuomotor integration, which are necessary for executive functioning, including working memory among young children.

In line with previous studies in typically developing children (84–86) and children with intellectual disabilities (71), our findings showed that gross motor skills ratings are associated with ratings of working memory in young children with ASD. While speculative in nature, a possible explanation for such findings might be the underlying cerebellar processes. The lateral zone of the cerebellum is intricately involved in regulating the motor activity of the whole body, namely the gross motor skills (87). Neuroimaging research has indicated the activation of the cerebellum during working memory tasks (88). Collectively, the present study differs from earlier research on children with ASD by offering a more nuanced understanding of the associations between fine and gross motor skills and working memory in both western and eastern countries.

### Motor skills and inhibition

The findings of this study indicated a significant association between parent ratings of fine and gross motor skills and inhibition ratings in children with ASD from the US. However, this association was not observed as significant among the Taiwanese children with ASD when adopting a stricter alpha level. This result mimics previous research on young children without ASD (49). Livesey et al. (49) utilized objective motor skill assessment (i.e., MABC) and Stroop test and indicated a significant association between motor skills and inhibitory control among 5–6 years old children without ASD. In addition to the explanation of co-activation of brain areas, this association between motor skills and inhibition might be posited from a behavioral learning perspective (89). For example, when children with ASD are in a learning environment, such as in preschool settings, they must pay attention and inhibit unrelated behaviors to properly demonstrate a fine motor task, such as writing, stringing beads, and manipulating objects. Inhibition is especially critical for young children, who may be more susceptible to environmental distractions in their surroundings. Evidence has suggested that inhibition emerges first during development in order for young children to ignore irrelevant stimuli and solve the problem (90). The ability to inhibit pre-potent responses might be an important first step in learning among young children.

While gross motor skills are generally considered to be associated with social skills or physical well-being (91), our results revealed that children with ASD’s gross motor skills ratings were significantly related to ratings of inhibition. This finding is consistent with previous cross-sectional (49) and longitudinal research (69). Wu et al. (69) have indicated that the early gross motor ability of 2-year-old infants predicts their inhibitory control at 3 years. In addition, evidence has suggested that the motor planning ability among children was associated with the capacity to inhibit a potent but irrelevant response (92). The item measured gross motor skills in ChAS-P included not only movement skills and ball skills but also included the item of maintaining balance while performing various activities (i.e., moving through obstacle courses), which likely involves certain aspects of motor planning. Further, this finding is aligned with the results of physical activity intervention studies. Research has revealed that physical activity involving gross motor exercise positively facilitates the processes of inhibitory control (93, 94). Our results highlight the importance of engaging in gross motor opportunities for children with ASD, given its association with inhibition.

The findings of the present study also revealed that ratings of fine motor skills had higher associations with EF ratings than gross motor skills. This result is aligned with research on children without ASD (95) as well as children with disabilities (72, 73). The difference observed in the link between gross motor skills and fine motor skills with EF may be attributable to the fact that fine motor skills exert a greater demand on the integrity of the cortical nervous system, specifically the frontoparietal network (96). Additionally, while the relationships between parent ratings of motor skills and EF were not significantly different in children with ASD from Taiwan and the US, the lower standardized beta coefficients were observed in Taiwanese children. This finding might be partially due to other influences of contextual factors. Evidence has indicated that both personal (e.g., comorbidity) and environmental factors (e.g., parenting practice) might affect motor and cognitive development (32, 97). However, information such as ADHD symptoms or parenting style is unavailable in this study. Thus, it is essential to acknowledge the influence of these factors on the association between ratings of motor skills and EF among young children with ASD.

Children with ASD often experience deficits in various domains that have long-term consequences. Motor skills difficulty puts an additional burden on the child and could impact their health, daily life, and social interactions considerably. Therefore, assessing and knowing the roles of fine and gross motor skills might help parents and professionals identify skills and programs that can be intervened early on in improvements of EF, which might also help provide these young children with ASD to reach their full potential in their developmental trajectory. The present findings provide some critical practical implications for parents and practitioners working with young children with ASD. Parents and practitioners should be aware of the specific relationship between both motor skills (i.e., fine and gross motor skills) and EF (i.e., working memory and inhibition). Such specific associations might indicate that early measurement of motor skills may be particularly beneficial for a child’s higher-order cognitive development, given the observed links between motor skills and EF. Neuroimaging evidence has indicated that the areas of the brain linked with more basic functions, including motor skills, mature first (98). Therefore, the development of early motor skills should be a priority. Parents and practitioners should provide and highlight both fine and gross motor opportunities in order to facilitate the EF of young children with ASD.

While this study has yielded meaningful findings with regard to the cross-cultural associations between parental rating of motor skills and EF of young children with ASD from Taiwan and the US, several limitations need to be considered. First, the severity level, IQ, and comorbidity status of the children with ASD from both countries were not reported in this study. Although various confounding variables were included in our analyses, it is important to mention that other variables that did not account for in the present study might have played a role, given that multiple systems would influence child development (99). Second, the motor skills and EF measurement of children with ASD were assessed via parental proxy report. Such subjective rating may be influenced by personal and cultural biases or beliefs, as well as prior experiences. In order words, parental perceptions might result in different bars in evaluating their child’s daily performance of motor skills and EF (32). It is worth noting that parent rating and performance-based measurement should not be used interchangeably as they capture different aspects; preferably using these two types of assessment in combination in the best-case scenario. Therefore, future research should utilize a combination of both parental reports and objective performance-based assessments of motor skills and EF to obtain more comprehensive and detailed information regarding the relationship between motor skills and EF among young children with ASD. Another limitation to note is the representativeness of our samples. The children with ASD included in this study were recruited through specific channels, which might not necessarily reflect a nationally representative sample of children with autism in the US and Taiwan. Consequently, while our findings provide meaningful insights, they may not be definitive. Instead, they should be viewed as an initial step in understanding potential cultural differences in parental ratings of motor skills and EF among children with ASD. This study lays the groundwork for further exploration in this area, but care should be taken in extrapolating the results to broader populations. Future cross-cultural studies would greatly benefit from recruitment strategies that ensure a more nationally representative sample, enhancing the generalizability and depth of the findings. Further, the current study did not recruit children without ASD as comparison groups, which might limit our ability to understand whether specific factors contribute to cross-cultural differences between children with and without ASD. In our analysis, it’s important to note that *R*^2^ statistics are influenced by the variability present in the dataset. Higher *R*^2^ values can result from datasets with greater variability, which may not necessarily imply a stronger model fit. Throughout the regression results section, we reported adjusted *R*^2^ values to account for the number of predictors in our models. It is also important to acknowledge the exploratory nature of our analyses, where we examined various relationships between motor skills and EF without a strict set of *a priori* hypotheses. This approach allowed us to explore potential associations comprehensively but also comes with the inherent risk of inflated Type 1 errors. To help account for this, we used an adjusted alpha level of 0.01 to control for the error rate across multiple tests. However, given the extensive nature of our analyses, we acknowledge the potential for inflated Type 1 errors, and readers should interpret the findings in light of this exploratory approach. While the present study provides valuable insights into the relationships under investigation, we also recognize the need for future research to confirm and replicate these findings. Lastly, the cross-sectional nature of the current study limits causal implications. Future studies should examine the motor and cognitive development of children with ASD using longitudinal design and assessments to gain more insight regarding how the relationship between motor skills and EF changes over time in the ASD population.

## Conclusion

This research is one of the first study to explore cross-cultural relationships between motor skills and EF of young children with ASD from Taiwan and the US. Overall results revealed that parent ratings of fine motor skills and gross motor skills were significantly associated with EF ratings in both working memory and inhibition among 4–6 years children with ASD from Taiwan and the US. Further, these associations between motor skills (i.e., fine motor and gross motor skills) and EF (i.e., working memory and inhibition) ratings were similar between the two countries. The present study is the important first step in understanding the relationships between motor skills and EF development. This study also sheds light on the importance of developing relevant initiatives and programs to create motor skills and EF intervention to build the early foundation for success later in school and in life among children with ASD.

## Data availability statement

The original contributions presented in the study are included in the article/supplementary materials, further inquiries can be directed to the corresponding author/s.

## Ethics statement

The studies involving humans were approved by the Institutional Review Board at Oregon State University. The studies were conducted in accordance with the local legislation and institutional requirements. The informed consent was obtained from the participants.

## Author contributions

M-CS: Conceptualization, Formal analysis, Investigation, Methodology, Project administration, Writing – original draft. MMM: Conceptualization, Writing – review & editing. WM: Conceptualization, Writing – review & editing. SL: Conceptualization, Writing – review & editing. MM: Conceptualization, Methodology, Supervision, Writing – review & editing.

## Footnotes

^1^ http://centiment.co

^2^ http://www.chexi.se
